# Supplementary material for: Resolving the full spectrum of human genome variation using Linked-Reads
Source: Genome Res. 2019 Apr;29(4):635–45. doi: 10.1101/gr.234443.118 (PMC6442396; doi:10.1101/gr.234443.118)
Supplement: Supplemental Material [file supp_gr.234443.118_Supplemental_longranger-2.2.zip › longranger-2.2.tar.bz2!/lr2.2-public/lib/stan/cmdstan/stan/doc/api/html/agrad_2fwd_2functions_2gamma__p_8hpp_source.html]

Stan: src/stan/agrad/fwd/functions/gamma\_p.hpp Source File


|  |  |
| --- | --- |
|  | Stan  2.5.0  probability, sampling & optimization |


- Main Page
- Related Pages
- Namespaces
- Classes
- Files

- File List
- File Members

All Classes Namespaces Files Functions Variables Typedefs Enumerations Enumerator Friends Macros Pages

- src
- stan
- agrad
- fwd
- functions

gamma\_p.hpp

Go to the documentation of this file.

1 #ifndef STAN\_\_AGRAD\_\_FWD\_\_FUNCTIONS\_\_GAMMA\_P\_HPP

2 #define STAN\_\_AGRAD\_\_FWD\_\_FUNCTIONS\_\_GAMMA\_P\_HPP

3

4 #include <stan/agrad/fwd/fvar.hpp>

5 #include <stan/meta/traits.hpp>

6 #include <stan/math/functions/gamma\_p.hpp>

7 #include <stan/prob/internal\_math.hpp>

8

9 namespace stan{

10

11  namespace agrad{

12

13  template <typename T>

14  inline

15  fvar<T>

16  gamma\_p(const fvar<T>& x1, const fvar<T>& x2){

17  using stan::math::gamma\_p;

18  using std::log;

19  using std::exp;

20  using std::pow;

21  using std::fabs;

22  using boost::math::tgamma;

23  using boost::math::digamma;

24

25  T u = gamma\_p(x1.val\_, x2.val\_);

26

27  T S = 0;

28  T s = 1;

29  T l = log(x2.val\_);

30  T g = tgamma(x1.val\_);

31  T dig = digamma(x1.val\_);

32

33  int k = 0;

34  T delta = s / (x1.val\_ \* x1.val\_);

35

36  while (fabs(delta) > 1e-6) {

37  S += delta;

38  ++k;

39  s \*= -x2.val\_ / k;

40  delta = s / ((k + x1.val\_) \* (k + x1.val\_));

41  }

42

43  T der1 = (u) \* ( dig - l ) + exp( x1.val\_ \* l ) \* S / g;

44  T der2 = exp(-x2.val\_) \* pow(x2.val\_, x1.val\_ - 1.0) / g;

45

46  return fvar<T>(u,x1.d\_ \* -der1 + x2.d\_ \* der2);

47  }

48

49  template <typename T>

50  inline

51  fvar<T>

52  gamma\_p(const fvar<T>& x1, const double x2){

53  using stan::math::gamma\_p;

54  using std::log;

55  using std::exp;

56  using std::pow;

57  using std::fabs;

58  using boost::math::tgamma;

59  using boost::math::digamma;

60

61  T u = gamma\_p(x1.val\_, x2);

62

63  T S = 0.0;

64  double s = 1.0;

65  double l = log(x2);

66  T g = tgamma(x1.val\_);

67  T dig = digamma(x1.val\_);

68

69  int k = 0;

70  T delta = s / (x1.val\_ \* x1.val\_);

71

72  while (fabs(delta) > 1e-6) {

73  S += delta;

74  ++k;

75  s \*= -x2 / k;

76  delta = s / ((k + x1.val\_) \* (k + x1.val\_));

77  }

78

79  T der1 = (u) \* ( dig - l ) + exp( x1.val\_ \* l ) \* S / g;

80

81  return fvar<T>(u, x1.d\_ \* -der1);

82  }

83

84  template <typename T>

85  inline

86  fvar<T>

87  gamma\_p(const double x1, const fvar<T>& x2){

88  using stan::math::gamma\_p;

89  using std::exp;

90  using std::pow;

91

92  T u = gamma\_p(x1, x2.val\_);

93

94  double g = boost::math::tgamma(x1);

95

96  T der2 = exp(-x2.val\_) \* pow(x2.val\_, x1 - 1.0) / g;

97

98  return fvar<T>(u, x2.d\_ \* der2);

99  }

100  }

101 }

102 #endif

stan::agrad::fabs

fvar< T > fabs(const fvar< T > &x)

**Definition:** fabs.hpp:18

stan::agrad::tgamma

fvar< T > tgamma(const fvar< T > &x)

**Definition:** tgamma.hpp:15

stan::agrad::pow

fvar< T > pow(const fvar< T > &x1, const fvar< T > &x2)

**Definition:** pow.hpp:17

stan::agrad::fvar

**Definition:** fvar.hpp:13

traits.hpp

fvar.hpp

stan::agrad::gamma\_p

fvar< T > gamma\_p(const fvar< T > &x1, const fvar< T > &x2)

**Definition:** gamma\_p.hpp:16

internal\_math.hpp

gamma\_p.hpp

stan::math::e

double e()

Return the base of the natural logarithm.

**Definition:** constants.hpp:86

stan::agrad::digamma

fvar< T > digamma(const fvar< T > &x)

**Definition:** digamma.hpp:16

stan::agrad::fvar::d\_

T d\_

**Definition:** fvar.hpp:16

stan::agrad::log

fvar< T > log(const fvar< T > &x)

**Definition:** log.hpp:15

stan::agrad::exp

fvar< T > exp(const fvar< T > &x)

**Definition:** exp.hpp:16

stan::math::gamma\_p

double gamma\_p(double x, double a)

**Definition:** gamma\_p.hpp:53

stan::agrad::fvar::val\_

T val\_

**Definition:** fvar.hpp:15


---

[ Stan Home Page ]

*© 2011–2014,
Stan Development Team.*
